# Factors affecting delays in oil and gas construction projects

**DOI:** 10.1038/s41598-025-31645-3

**Published:** 2025-12-27

**Authors:** Medhat Youssef, Ahmed Gab-Allah, Omar Hagras, Ibrahim Bahader

**Affiliations:** Construction Engineering and Utilities Department, Faculty of Engineering, Zagazig University, Zagazig, Al-Sharqia Egypt

**Keywords:** Construction delay causes, Managing construction delay, Egyptian oil and gas construction projects, Management practices of delay, Energy science and technology, Engineering

## Abstract

Ensuring the timely completion of construction projects is vital for overall efficiency. This study aims to identify, classify, and rank the main factors of delay in Egyptian oil and gas construction projects related to project stakeholders. A mixed-methods approach was used to Sharkia collect and analyze both quantitative and qualitative data. Data was gathered from 71 sector respondents. Expert interviews refined the delay factors from the literature to 20 for contractors, 17 for owners, and 13 related to consultants. Financial difficulties, late payments to suppliers and subcontractors, and supplier material delivery delays were the significant causes related to the contractor. Delays associated with owners came from ineffective contractor selection and government regulations. Consultants contributed to delays in finalizing drawings and designs. To provide a deeper understanding of the interrelationships among these factors and their collective effect on project delay, a structural model was developed and evaluated. The structural model demonstrated that deficiencies in project planning, design management, and owner decision-making collectively contribute to delays. This provides a basis for developing integrated management approaches to mitigate future delays and improve project performance in Egypt’s oil and gas construction sector.

## Introduction

Delays present a global challenge in construction projects, with each project exhibiting unique characteristics influenced by various factors and external constraints. This issue is particularly true for oil and gas construction projects, which typically involve long implementation periods and substantial financial investments^1,2^. Such projects demand a wide range of expertise, interdisciplinary cooperation, and the engagement of international experts and local teams^3^. Furthermore, the participation of multiple nationalities in the design and execution adds more complexity to these projects^4^. Additionally, the success of these projects largely depends on thorough planning and meticulous preparation during the initial stages^5^.

Numerous previous studies have investigated the causes of delays in construction projects in general and proposed various solutions to mitigate their negative impact on project timelines, budgets, and stakeholder relationships. Although extensive research exists on delays and their contributing factors, studies specifically addressing delays in oil and gas construction projects remain limited.

Most existing research focuses on the factors of delays in projects within oil- and gas-dependent countries, including those in the Gulf region, Libya, Iran, and the United States. However, there is a lack of research on the causes and consequences of delays in oil and gas construction projects in developing countries, such as Egypt, where the demand for energy resources is critical and rapidly growing. Hence, this study explores these challenges.

Egypt is one of Africa’s most energy-consuming developing countries^6^. It currently ranks third in population on the continent, highlighting the need for significant attention to energy projects to meet its diverse energy demands. While numerous studies in Egypt have examined this issue in public, private, infrastructure, and industrial projects, no study has exclusively focused on addressing the phenomenon of delays in Egyptian oil and gas construction projects. This study aims to fill this gap by identifying, classifying, and evaluating the key causes of construction delays in Egypt’s oil and gas sector on an individual basis. It also proposes mitigation strategies to address these factors. Furthermore, an integrated analytical framework combining Exploratory Factor Analysis (EFA), and Structural Equation Modeling (SEM) is employed to examine the delay causes collectively as interrelated groups and to assess their overall impact on project timelines.

## Literature review

### Causes of delay in oil and gas construction projects

Delays in oil and gas construction projects have been a significant concern worldwide due to their economic significance and substantial financial investments. These delays can have direct implications for national income and project feasibility. Various studies have identified key factors of delays across different countries.

In Iranian oil and gas construction projects, a lack of skilled labor, unclear contract terms, political challenges, inadequate planning, payment challenges, material procurement issues, lengthy approval processes, ineffective early negotiations, and contractor breaches significantly impact project timelines^7–10^. Although these studies provided a relatively comprehensive list of influencing factors, they focused mainly on the descriptive dimension of the factors without linking them in terms of their frequency or intensity of influence on temporal performance.

Two studies in Indonesia have identified several key challenges contributing to construction delays, including contractor performance, logistics, government approvals, societal and environmental concerns, and commercial factors. Additionally, financial constraints faced by contractors were found to be a significant factor delaying EPC projects^11,12^. These reasons collectively impact project efficiency.

Under financial constraints for both contractors and owners, a study on project delays in Oman showed that the main factors contributing to delays include owner dependence on the lowest financial offer and financial problems faced by the contractor^13^. Similarly, within the context of owner financial issues, a study in Egypt recommended that the owner must provide the cash flow for the project before selecting a general contractor^14^.

In a different national context, delays in Iraq’s petroleum and gas industry have resulted from low bidding by contractors, financial instability, poor scheduling, and inadequate planning^15^. In the case of Yemen, internal risks played a critical role, followed by design changes during construction, government instability, inaccurate cost estimation, political uncertainty, and conflict^16^. These two studies reflect the specificity of the political and economic environments in the region; however, most of them provided only descriptive analyses without statistically grouping the factors into specific categories.

In Thailand, delays in oil and gas platform projects were mainly caused by unskilled labor supplied by vendors and ineffective site management by contractors^17^. Research on Malaysian oil and gas construction projects categorized the contributing factors into six main groups: owner-related, contractor-related, engineering-related, external influences, project-specific issues, and resource constraints^18^.

At the regional level, studies within the Gulf Cooperation Council (GCC) countries have also identified several common factors, including poor planning and scheduling by contractors, subcontractor-related issues, material delivery delays, ineffective communication among stakeholders, and challenges in vendor coordination during procurement^19^. In the UAE, key delay factors include late procurement of long-lead items, material delivery disruptions, insufficient technical expertise, weak project management, and a shortage of qualified engineers^20^. Addressing these challenges through improved planning, efficient resource allocation, and strong oversight is essential for enhancing construction efficiency in the oil and gas sectors.

In Saudi Arabia, major delay factors in oil and gas projects have been identified as poor planning, unclear project scope during the bidding phase, design modifications requested by the owner, and design errors^21^. For liquefied natural gas (LNG) projects, common causes of delays include contract modifications, procurement disruptions, design changes, slow decision-making, and payment delays, all of which significantly impact project timelines^22^. These challenges require enhancement of project planning, clear scope definition, efficient procurement strategies, and effective communication to minimize delays.

Research on oil and gas pipeline projects across various countries, including Iran, Bahrain, and Australia, has identified common delay factors such as material supply shortages, unrealistic scheduling, land acquisition issues, modification orders, challenges in contractor selection, scope variations, late material deliveries, inadequate planning, design errors, frequent design modifications, and ineffective communication among stakeholders^23–25^. Despite regional differences, these results indicate that pipeline projects worldwide face recurring delay challenges, emphasizing the need for effective strategies to mitigate delays.

### Delay mitigation practices and benefits

Many studies have emphasized the importance of addressing delay causes through effective mitigation strategies. A study conducted in Jordan indicated that early identification and treatment of the leading causes of delays can significantly contribute to reducing their impact on project schedules, reducing costs, and strengthening stakeholder relationships^26^. Developing effective strategies and proactive plans is therefore crucial to mitigate potential risks that affect the project’s budget, timeline, and quality^16^. Among the decisive factors in minimizing delays and their impacts are the selection of qualified contractors and consultants, along with the implementation of value engineering and cost reduction measures^27^. Similarly, delays in Vietnam’s petrochemical industry highlight the necessity of employing experienced contractors with strong technical and managerial capabilities to ensure timely completion^28^. Additionally, a study investigating the role of new technology in mitigating delays highlighted 73 major reasons for delays, among which 25 factors were attributed to the failure to adopt modern technology and reliance on traditional construction techniques^29^. Prior research has focused on evaluating the significance of delay causes based on expert and practitioner opinions, institutional and organizational contexts, without adopting comprehensive analytical approaches to better understand the factors causing delays and their interrelationships, often neglecting the analysis of their frequency or individual impact on project timelines. Moreover, most current studies have not employed statistical techniques to examine the interrelationships among delay factors as cohesive groups, especially within a developing-country context.

To date, no study has been found that specifically examines delays in oil and gas construction projects within the Egyptian context. The present study aims to fill this gap by employing a holistic approach that integrates field data with comprehensive statistical analysis.

## Study methodology

This study employs a mixed-methods exploratory sequential design, combining quantitative and qualitative data collection to provide a comprehensive understanding of the research problem^30^. The quantitative aspect focuses on collecting digital data through closed questions in the questionnaire and statistically analyzing it to identify patterns and relationships in four stages.

In the first stage, the delay factors identified in previous studies were refined and prioritized by experts to ensure contextual relevance and accuracy. The quantitative evaluation was conducted individually using the Relative Importance Index (RII) and Critical Ranking methods to prioritize their significance.

In the second stage, the factor groups were derived by conducting an Exploratory Factor Analysis (EFA) using the raw item-level responses for the three axes (importance, impact, and frequency) within the SPSS program.

In the third stage, Partial Least Squares Structural Equation Modeling (PLS-SEM) using Smart-PLS was employed to examine the relationships between the four derived groups and a latent variable representing the overall delay.

Based on the statistical results, emphasis was placed on identifying the most critical factors contributing to project delays in the oil and gas sector. This dual analytical approach enabled a comprehensive understanding of how specific factors contribute individually and collectively to overall project delays.

**Fig. 1:**
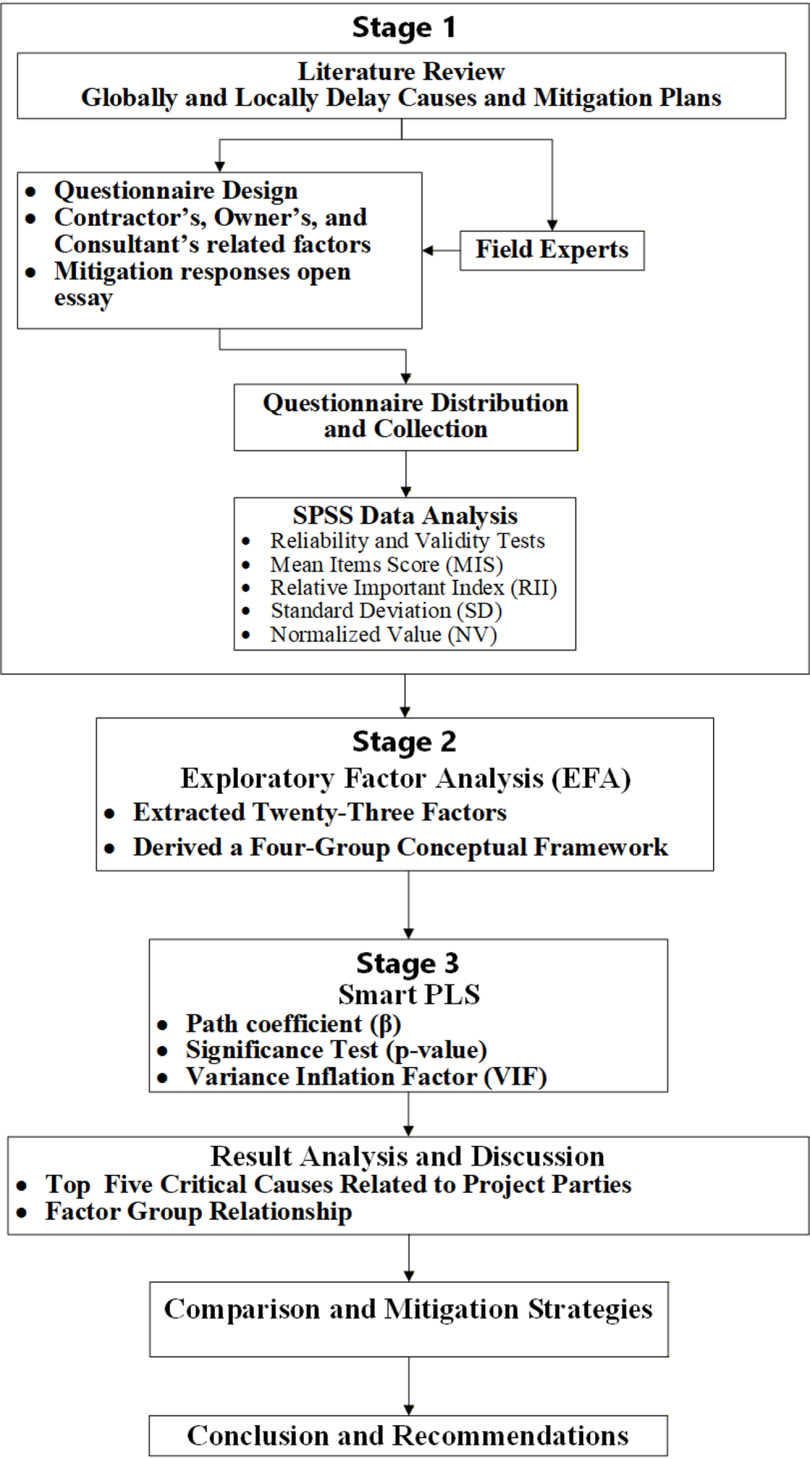
Study methodology.

To develop practical evidence-based solutions, a set of proposed strategies to reduce these factors has been identified. These strategies were derived from a comprehensive review of previous studies and then compared with the recommendations and solutions adopted by survey participants to assess the compatibility between theoretical literature and the applied visions of workers in the petroleum projects sector in Egypt. This comparative analysis contributes to the formulation of radical strategies to address delays within the scope of petroleum construction and to enhance the efficiency of implementation by reducing the occurrence of these causes in the future. The study methodology is illustrated in Fig. 1.

### Research population and sample size

The target population for this study comprises three stakeholder groups within Egypt’s oil and gas sector. These include public and private sector entities, with significant input from the experts of the Egyptian General Petroleum Corporation (EGPC), who contributed to reviewing and refining the research questionnaire to ensure its relevance and clarity. The groups are owners, contractors, and consultants.

For statistical significance and to achieve a sufficient and reliable sample size, it was calculated using the formula proposed by Bartlett^31^, which depends on the first and second Cochran (1977) equations:

1 $$\:{\mathrm{n}}_{0}=\frac{{\mathrm{K}}^{2}\mathrm{*}P\mathrm{*}Q}{{S}^{2}}$$

Where $\:{\mathrm{n}}_{0}$ = appropriate sample size, K = 1.645 for a 90% confidence level, P: Proportion of.

the respondents stating that this factor is effective = 50%, Q: Proportion of the respondents stating that this factor isn’t effective= (Q = 1-P) Where (p)* (q) = estimate of variance = 0.25, S = acceptable margin of error = 10%.

2 $$\:{\mathrm{n}}_{1}=\frac{{\mathrm{n}}_{0}}{1+\frac{{\mathrm{n}}_{0}}{z}}$$

Where $\:{\mathrm{n}}_{1}$ represents the required sample size, Z is the population size.

According to the Egyptian Central Agency for Public Mobilization and Statistics, the number of employees in the petroleum sector in Egypt reached 156,000 in 2021. This reflects the relatively small sample size of the oil and gas construction sector compared to that of the general construction industry. By applying these equations and substituting the predefined variables, the sample size is 68 individuals.

### Questionnaire design

**Fig. 2:**
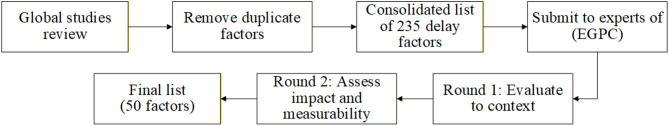
Refinement stages of delay factors.

A comprehensive global review of previous studies on delay factors identified a wide range of causes attributed to the three primary project stakeholders. Some factors derived from previous research were conceptually similar or overlapping in meaning, even though they were expressed under different names; therefore, these items were removed before being submitted to experts. The consolidated preliminary list comprised 110 factors for contractors, 68 for owners, and 57 for consultants. The list was reviewed by experts from the Egyptian General Petroleum Corporation (EGPC) through two evaluation rounds to assess the relevance and applicability of these factors to Egyptian petroleum projects. Figure 2 illustrates the refinement process, which reduced the total number of delay factors from 235 to 50.

Based on the process of revising factors, the list was refined and adapted to 20 delay factors specific to contractors, 17 delay factors related to owners, and 13 delay factors for consultants, as shown in Table 1.

**Table 1:** Factors of delay related to stakeholders.

Category	Code	Causes of delay
Contractor	F1	Poor scheduling and outdated technology.
	F2	Supplier delays in delivering materials to the contractor.
	F3	Miscommunication with project parties.
	F4	Ambiguity in the scope of work.
	F5	Financial issues.
	F6	Inappropriate construction methods employed.
	F7	Unbalanced tender submitted by the contractor.
	F8	Failure to adhere to Occupational Safety and Health regulations.
	F9	Delays in payment to subcontractors and suppliers.
	F10	Shortage of skilled labor within the team.
	F11	Inadequate selection of subcontractors and suppliers.
	F12	Errors by the contractor and subcontractors leading to rework.
	F13	Inadequate inspection and testing of equipment and materials prior to supply.
	F14	Low productivity of the work team
	F15	Lack of raw materials.
	F16	Ineffective monitoring and feedback methods.
	F17	Poor coordination among subcontractors.
	F18	Changes in teamwork and staff.
	F19	Incentives for early termination, penalties for delays, and consequential damages.
	F20	Incorrect understanding of the technical specifications of the project items.
Owner	F21	Financial issues.
	F22	Requests for changes.
	F23	Ambiguity in the scope of work.
	F24	Poor contractor selection.
	F25	Government regulations and laws.
	F26	Delays in the delivery of the site to the contractor.
	F27	Late review and approval of documents.
	F28	Lack of communication with various parties involved in the project.
	F29	Lack of experience of employees with the project scope and requirements.
	F30	The owner failed to provide materials and equipment as per the contract.
	F31	Frequent changes in management staff.
	F32	Lengthy decision-making time.
	F33	Failure to consider maintainability during the design phase.
	F34	Delay in approval of engineering drawings.
	F35	Delays in approval of supplied raw materials.
	F36	Suspension of work.
	F37	Inappropriate choice of consultant.
Consultant	F38	Lack of knowledge among staff regarding project quality assurance, control and specifications.
	F39	Ineffective participation.
	F40	Delays in preparing the design, final drawings, and obtaining approval.
	F41	Late receipt of project items.
	F42	Lack of communication with project stakeholders.
	F43	Discrepancies between the drawings and technical specifications in the contract.
	F44	Design errors.
	F45	Deficiencies in the project feasibility studies
	F46	Ambiguous formulation of the agreed-upon scope of work with the owner
	F47	Lack of coordination between designs from different disciplines.
	F48	Inappropriate selection of contractor.
	F49	Delays in approving significant changes to the scope of work.
	F50	Ambiguity in the technical specifications of project items.

The refined delay factors formed the foundation for the final design of the questionnaire, which was prepared in both Arabic and English languages before being distributed to participants. The questionnaire is structured into three main sections: the first outlines the study’s objectives, the second provides definitions of key terms, and the third offers instructions on how to fill out the questionnaire accurately. Furthermore, the questionnaire is divided into several categories: Personal Information, Ordinal data, and Multiple-choice questions that allow for a broader range of responses to be captured. The questionnaire includes open-ended questions to gather more detailed insights from participants.

The final section of the questionnaire focuses on practices, recommendations, solutions, and potential measures to mitigate the negative impacts of delays in the construction of oil and gas projects in Egypt.

### Personal and ordinal data

The questionnaire used in this study included both closed and open-ended questions. The closed-ended questions focused on gathering personal and professional information from respondents, including their name, academic qualifications, years of experience, the approximate number of oil and gas projects they have participated in, and their roles in the sector (owners, contractors, or consultants) in the context of oil and gas projects in Egypt.

In addition to identifying the causes of delays in these projects, the questionnaire explored two additional critical dimensions: the degree of impact that these causes have on project schedules and the frequency with which these delay factors occurred in the respondents’ previously executed projects. This approach allowed for a more comprehensive understanding of the most significant, impactful, and frequent delay factors in the construction of oil and gas projects in Egypt. To measure these dimensions, the second section of the questionnaire employed a Likert scale, a widely used tool in multi-party surveys for assessing perceptions and attitudes^32^. The Likert scale consisted of five response categories for each of the three dimensions—importance, frequency, and impact. This scale allowed for the collection of quantitative data, facilitating an analysis of the relative importance.

### Data collection and analysis

One hundred questionnaires were distributed with the aim of obtaining at least sixty-eight responses. Methods for accessing the sample included in-person interviews, corporate websites, social media platforms, electronic surveys, and phone calls. Seventy-one valid responses were collected, representing the main parties involved in oil and gas projects in Egypt: forty-eight owners, eighteen contractors, and five consultants.

It is noted that the sample distribution is unbalanced, with owners comprising the largest percentage, while consultants represent the lowest category. This irregular distribution is one of the limitations of the data collection process, due to the difficulty of achieving balanced participation from all parties in the oil and gas sector. This imbalance may affect the generalizability of the results and explain the divergence of views.

### Reliability and validity tests

Before conducting the data analysis, Cronbach’s alpha coefficients were calculated to evaluate the internal consistency of the questionnaire data. This statistical measure assesses the reliability of a scale by determining the consistency of responses across its items, particularly those measured using a Likert scale. Cronbach’s alpha is widely used in research to verify the reliability and validity of instruments designed to capture subjective perceptions and opinions. The formula for calculating Cronbach’s alpha is as follows:

3 $$\:{\upalpha\:}=\frac{\mathrm{K}}{\mathrm{K}-1}(1-\frac{\sum\:{\mathrm{V}}_{\mathrm{i}}}{{\mathrm{V}}_{\mathrm{t}\mathrm{o}\mathrm{t}}})$$

Where K represents the number of items (questions) in the scale, Vi is the variance of each individual item, and V_tot_ is the total variance of the entire scale (all items combined).

Table 2 presents the calculated Cronbach’s Alpha coefficients, the Kendall coefficient of compatibility (W), the Kaiser-Meyer-Olkin values (KMO), and the Bartlett’s test of sphericity. All Cronbach’s Alpha values exceed 0.7, indicating a high level of internal consistency and reliability in the collected data^33^. Kendall’s coefficient (W), ranging from 0 (no agreement) to 1 (complete agreement), was used to evaluate respondents’ agreement^34^. The results showed that Kendall’s coefficient (W) values were low across all dimensions of the questionnaire, indicating a limited level of consensus among respondents regarding the three study dimensions. This limited agreement can be attributed to the differing viewpoints, priorities, variations in roles, responsibilities, and perceptions among stakeholders in construction projects^35^.

**Table 2:** Cronbach’s Alpha, kendall’s coefficient, KMO, and sphericity coefficient Values.

Category	Dimension	Number of clauses	Cronbach’s Alpha	Kendall’s Coefficient (W)	KMO Value	Sphericity		
						chi-square	Df	Sig.
Contractor	Importance	20	0.934	0.054	0.894	932.42	190	0.001
	Impact	20	0.931	0.070	0.875	892.6	190	0.001
	Frequency	20	0.925	0.066	0.885	817.50	190	0.001
Owner	Importance	17	0.927	0.084	0.837	719.13	136	0.001
	Impact	17	0.971	0.081	0.785	580.32	136	0.001
	Frequency	17	0.883	0.118	0.791	518.33	136	0.001
Consultant	Importance	13	0.965	0.013	0.921	948.02	78	0.001
	Impact	13	0.942	0.007	0.916	663.95	78	0.001
	Frequency	13	0.936	0.014	0.879	623.81	78	0.001
Overall	Overall	150	0.984	0.106	0.720	695.63	36	0.001

The Kaiser-Meyer-Olkin test showed a total value of 0.720 in overall responses, indicating a moderate level of sampling adequacy and confirming the suitability of the data for factor analysis^36^. Additionally, Bartlett’s test of sphericity revealed a large chi-squared value (approximately chi-squared = 695.632, degrees of freedom = 36, p-value = 0.001), demonstrating significant correlations between variables^37^. These results provide a basis for extracting the underlying factors and analyzing the structural relationships within the three dimensions examined from the perspective of all stakeholders.

### Materials and methods

The study was conducted through personal questionnaires with the full informed consent of all participants. Consent was explicitly stated within the questionnaire itself. All personal data collected was used only for scientific research purposes.

### Ethical and compliance approval

The Faculty of Engineering at Zagazig University in Egypt approved this study, confirming compliance with all institutional and national standards for research involving human participants. Written informed consent was obtained from all participants prior to their enrolment in the study. Participation was voluntary, and all procedures were conducted in accordance with recognized ethical standards for research involving human participants. Participants were informed about the purpose of the study and were assured that their responses would remain confidential. Official documents confirming ethical consent are available upon request.

## Results and discussion

### Demographic information

The current study included 71 participants, which is comparatively larger than the sample sizes in similar studies (59, 23, and 42) reported by^19,24^, and^4^, respectively. This may strengthen the validity and reliability of the results. Participants represented both public and private sector organizations covering a variety of job roles, including Site Engineers, Project Managers, Cost Managers, Executives, Senior Management staff of oil companies, Industry specialists, and other related roles. Regarding education, over 75% of respondents hold a bachelor’s degree, 8.4% a master’s degree, and 15.5% represent other qualifications, including diplomas. In terms of experience, 48% of participants had more than 15 years of professional experience, while 37% had between 10 and 15 years. Despite efforts to maximize participation, the response rate fell short of initial expectations, most likely due to the limited size of the target population. Table 3 details respondents’ roles, experience levels, educational qualifications, and types of projects they implemented.

**Table 3:** Demographic information.

Respondents		Frequency	Percentage (%)	Cumulative (%)
Category	Site engineer	20	28.17	28.17
	Project manager	15	21.13	49.30
	Head manager	5	7.04	56.34
	Consultants Engineer	5	7.04	63.38
	Executive director	8	11.27	74.65
	Contractor	18	25.35	100
Academic qualification	Bachelor’s degree	53	74.65	74.65
	Master’s degree	6	8.45	83.10
	PhD	1	1.41	84.51
	Other qualifications	11	15.49	100
Experience	> 15 year	34	47.88	47.88
	10:15	26	36.62	84.50
	5:10	8	11.27	95.77
	< 5 year	3	4.23	100
Types of projects	Oil	60	84.50	84.50
	Gas	11	15.50	100

### Causes of delay: importance, impact and frequency ranking

Numerous previous studies that utilized Likert scale data have applied the Relative Importance Index (RII) as an analytical technique to rank and identify the most important causes of delays in construction projects^32^. Therefore, the factors causing delays in construction projects in this study were identified by analyzing questionnaire responses using the Relative Importance Index (RII) method, which provides a unified scale for classifying the causes of delays. The weights were distributed according to the levels of agreement of the main stakeholders: contractors, owners, and consultants. The RII method offers a distinct advantage by providing a standardized index ranging from 0 to 1, facilitating direct comparisons across different variables. This method provides a powerful quantitative tool to identify the most important factors influencing delay based on the opinions of the study participants. Consequently, the RII formula proposed by^38^ was adopted for this analysis:

4 $$\:\mathrm{R}\mathrm{I}\mathrm{I}=\mathrm{A}\times\:\mathrm{N}{\Sigma\:}\left(\mathrm{W}\right)$$

Where: $\:\mathrm{W}$ = Weight assigned to each factor by respondents (e.g., on a scale of 1 to 5), $\:\mathrm{A}$ = Maximum possible weight (e.g., 5 for a 5-point Likert scale), and $\:\mathrm{N}$ = Total number of respondents.

The weights derived using this formula were proportionally distributed across five levels of respondent agreement identified by the three main stakeholder groups: contractors, owners, and consultants.

The survey examined the impact of these factors on projects execution using a Likert scale, and the collected data were analyzed using SPSS (version 26). Furthermore, the frequency of these causes was analyzed to identify the most common factors. Table 4 provides an overview of the most significant delay causes, evaluated based on their importance, impact, and frequency. These causes are ranked using Critical Rank based on their RII, reflecting the perspectives of respondents.

### Common causes in terms of importance, impact, and frequency

The analysis evaluated three terms: importance, impact, and frequency to ensure alignment with the study’s objective. Based on the results, the common causes that received the highest rating stand out with complete agreement among the respondents, individually and collectively: F5, F9, F2, F25, and F40. These causes can be classified under a hypothetical label from the study’s analysis as “Factors with Full Stakeholder Consensus”. Notably, the same factors that achieved full consensus among respondents in this study were also ranked first in some previous research on project delays, as presented in Table 5.

### Development and validation of the structural model

#### Exploratory factor analysis

The data were analyzed using exploratory factor analysis (EFA) to verify the underlying structure of latent variables. This method aimed to reduce the observed variables into a smaller set of factors based on the eigenvalue greater than one criterion proposed by Kaiser (1960). A screen plot was also employed to identify the point at which intrinsic factors could be distinguished from those with limited explanatory power. By combining numerical and visual criteria, the identifications of latent factors became more reliable. The suitability of the data for factor analysis was assessed using the Kaiser-Meyer-Olkin (KMO) measure and Bartlett’s test of Sphericity. A factor loading criterion of 0.40 was adopted as the minimum criterion for retaining variables within each factor^39^. Based on these criteria, a four-group conceptual framework was developed, which included twenty-three delay factors that represent the most significant, influential, and frequent factors in the interpretation of data variability. Variables with a factor loading below 0.40 or that did not demonstrate sufficient stability across more than one factor were excluded from the analysis. The factor loadings ranged from 0.445 to 0.850, indicating associations ranging from weak to strong. Low-load indicators (F32) were retained due to their theoretical and practical relevance in explaining the delay phenomenon^40^. Table 6 presents all latent variables, indicators, load values, and academic interpretations.

**Table 4:** RII, normalized value and critical ranking of the Importance, Influence, and frequency of delay Factors.

CODE	IMPORTANCE			EFFECT			FREQUENCE		
	RII	*N*.V	C.*R*	RII	*N*.V	C.*R*	RII	*N*.V	C.*R*
F1	0.76	0.75	3	0.78	0.75	2	0.69	0.81	4
F2	0.74	0.66	5	0.75	0.60	3	0.69	0.83	3
F3	0.68	0.33	15	0.70	0.35	12	0.62	0.37	14
F4	0.65	0.17	19	0.70	0.35	11	0.59	0.13	18
F5	0.80	1.00	1	0.83	1.00	1	0.70	0.91	2
F6	0.66	0.20	18	0.66	0.16	17	0.58	0.11	19
F7	0.71	0.47	11	0.70	0.32	13	0.63	0.43	12
F8	0.67	0.27	16	0.66	0.15	18	0.62	0.35	15
F9	0.77	0.84	2	0.75	0.60	4	0.72	1.00	1
F10	0.62	0.00	20	0.63	0.00	20	0.57	0.00	20
F11	0.75	0.69	4	0.73	0.50	7	0.67	0.67	6
F12	0.70	0.45	12	0.71	0.38	9	0.63	0.44	11
F13	0.66	0.22	17	0.67	0.19	16	0.60	0.22	16
F14	0.72	0.56	9	0.73	0.51	6	0.66	0.63	7
F15	0.74	0.64	6	0.74	0.56	5	0.68	0.76	5
F16	0.74	0.63	7	0.70	0.37	10	0.65	0.56	8
F17	0.70	0.45	13	0.68	0.24	15	0.65	0.56	9
F18	0.70	0.42	14	0.65	0.10	19	0.64	0.50	10
F19	0.71	0.47	10	0.68	0.25	14	0.63	0.41	13
F20	0.73	0.58	8	0.72	0.46	8	0.59	0.19	17
F21	0.72	0.59	8.00	0.74	0.67	8.00	0.60	0.38	10
F22	0.76	0.76	3	0.78	0.88	3	0.71	0.78	2
F23	0.68	0.38	14.00	0.70	0.48	14.00	0.59	0.33	12
F24	0.75	0.70	6.00	0.77	0.85	4.00	0.66	0.61	5
F25	0.82	1.00	1	0.81	1.00	1	0.77	1.00	1
F26	0.63	0.19	15.00	0.66	0.29	15.00	0.58	0.29	14
F27	0.73	0.61	7.00	0.75	0.73	7.00	0.66	0.61	6
F28	0.68	0.40	13.00	0.71	0.55	13.00	0.58	0.31	13
F29	0.71	0.51	11.00	0.73	0.65	9.00	0.62	0.44	8
F30	0.59	0.00	17.00	0.59	0.00	17.00	0.50	0.00	17
F31	0.63	0.15	16.00	0.64	0.23	16.00	0.55	0.20	16
F32	0.77	0.81	2	0.79	0.92	2	0.68	0.69	3
F33	0.72	0.56	9.00	0.71	0.55	12.00	0.64	0.51	7
F34	0.76	0.74	4	0.76	0.80	5	0.68	0.66	4
F35	0.72	0.55	10.00	0.73	0.65	10.00	0.61	0.43	9
F36	0.68	0.41	12.00	0.71	0.56	11.00	0.56	0.23	15
F37	0.75	0.71	5.00	0.76	0.79	6.00	0.59	0.35	11
F38	0.72	0.00	12.00	0.75	0.48	5.00	0.61	0.41	8
F39	0.75	0.50	7.00	0.76	0.71	2	0.62	0.64	4
F40	0.77	1.00	1	0.78	1.00	1	0.64	1.00	1
F41	0.74	0.25	8.00	0.74	0.38	7.00	0.61	0.55	6
F42	0.74	0.25	9.00	0.74	0.33	9.00	0.60	0.36	10
F43	0.75	0.50	6.00	0.74	0.24	11.00	0.59	0.23	11
F44	0.73	0.19	10.00	0.75	0.57	4.00	0.58	0.00	13
F45	0.73	0.06	11.00	0.73	0.19	12.00	0.61	0.50	7
F46	0.75	0.62	4.00	0.74	0.38	7.00	0.62	0.59	5
F47	0.76	0.87	3	0.76	0.62	3	0.63	0.77	2
F48	0.75	0.56	5.00	0.75	0.43	6.00	0.60	0.36	9
F49	0.77	0.94	2	0.74	0.29	10.00	0.63	0.73	3
F50	0.72	0.00	12.00	0.72	0.00	13.00	0.59	0.23	12

**Table 5:** Factors with full consensus comparative with previous Studies.

Category	Rank	Code	Causes of delay	Previous studies
Contractor	1	F 5	Contractor financial issues.	^18^and^41^
	2	F 9	Delays in payment to subcontractors and suppliers.	^17^
	3	F2	Supplier delays in delivering materials to the contractor.	^23,]^^41,]^^20^ and^17^
Owner	1	F 25	Government regulations and laws.	^4^and^41^
Consultant	1	F40	Delay in preparing the design, final drawings, and obtaining approval.	^23^and^42^

**Table 6:** Factor loading of latent variables.

Latent Variable ( LV )	Factor	Factor Loading	Interpretation
Design Management and Coordination Issues	F32	0.583	Acceptable
	F39	0.823	Very Strong
	F40	0.843	Very Strong
	F41	0.822	Very Strong
	F46	0.717	Good
	F47	0.572	Acceptable
	F48	0.773	Good
	F49	0.850	Very Strong
Financial and Resources Constraint	F2	0.766	
	F5	0.728	Good
	F9	0.659	Acceptable
	F11	0.558	Acceptable
	F14	0.558	Acceptable
	F15	0.746	Good
	F35	0.545	Acceptable
Project Planning and Regulatory issues	F1	0.781	Good
	F25	0.527	Acceptable
	F47	0.505	Acceptable
	F24	0.676	Good
	F34	0.693	Good
Owner-related Decision and Approval Delays	F22	0.542	Acceptable
	F27	0.810	Very Strong
	F32	0.445	low

**Fig. 3:**
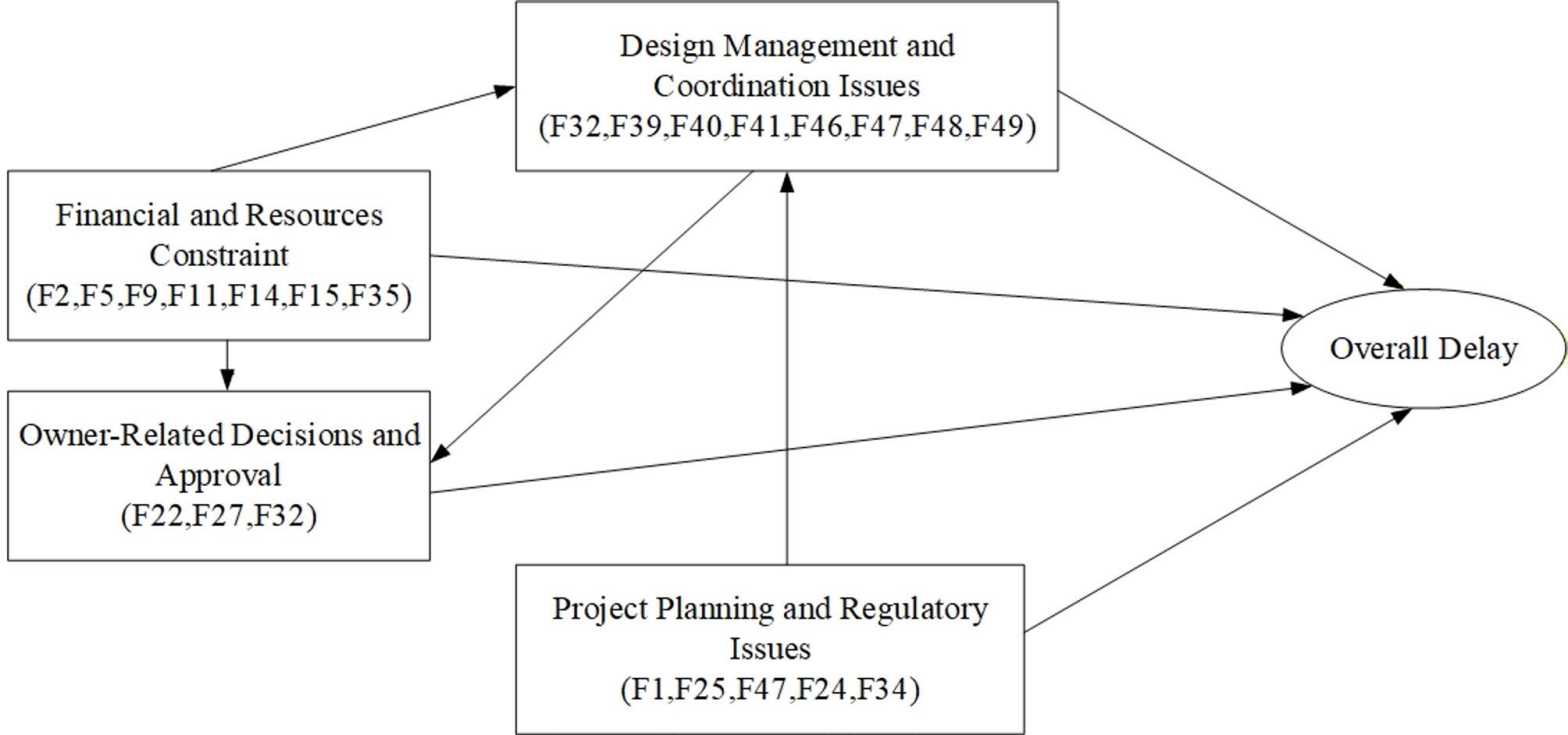
The empirically developed conceptual framework.

Based on the EFA results, a conceptual framework was developed to illustrate the relationships among the derivative delay factor groups. Figure 3 presents the developed conceptual framework.

#### Structural equation modelling

The variance-based Structural Equation Model (PLS-SEM) was developed using SmartPLS software to analyze the interrelationships among the four groups identified through Exploratory Factor Analysis (EFA), as well as the relationships between these groups and a formative latent variable representing Overall Delay. The composite scores of the four groups were utilized to define and reflect their contribution to the Overall Delay construct. The path coefficients and their statistical significance were assessed using *t*-statistics and *p*-values. The results indicated that relationships among the groups are direct and statistically significant, influencing Overall Delay^43^, confirming that these groups constitute the key explanatory dimensions of delay in Egyptian petroleum construction projects. Table 7 summarizes the path coefficients along with their respective statistical values.

**Table 7:** Path coefficients and significance levels of the structural model.

Independent variable (predictor )	Dependent variable ( outcome)	β	VIF	*p*-value
Design Management and Coordination Issues	Overall Delay	0.34	2.69	0.001
Design Management and Coordination Issues	Owner-related Decision and Approval Delays	0.57	1.57	0.001
Financial and Resources Constraint	Design Management and Coordination Issues	0.05	2.74	0.001
Financial and Resources Constraint	Overall Delay	0.39	2.77	0.001
Financial and Resources Constraint	Owner-related Decision and Approval Delays	0.24	1.57	0.001
Owner-related Decision and Approval Delays	Overall Delay	0.14	2.31	0.001
Project Planning and Regulatory issues	Design Management and Coordination Issues	0.69	2.75	0.001
Project Planning and Regulatory issues	Overall Delay	0.25	3.93	0.001

Results show that Project Planning and Regulatory issues are strongly linked to Design Management and Coordination (β = 0.69, *p* < 0.001), confirming that poor planning and organizational procedures increase design- related problems.

**Fig. 4:**
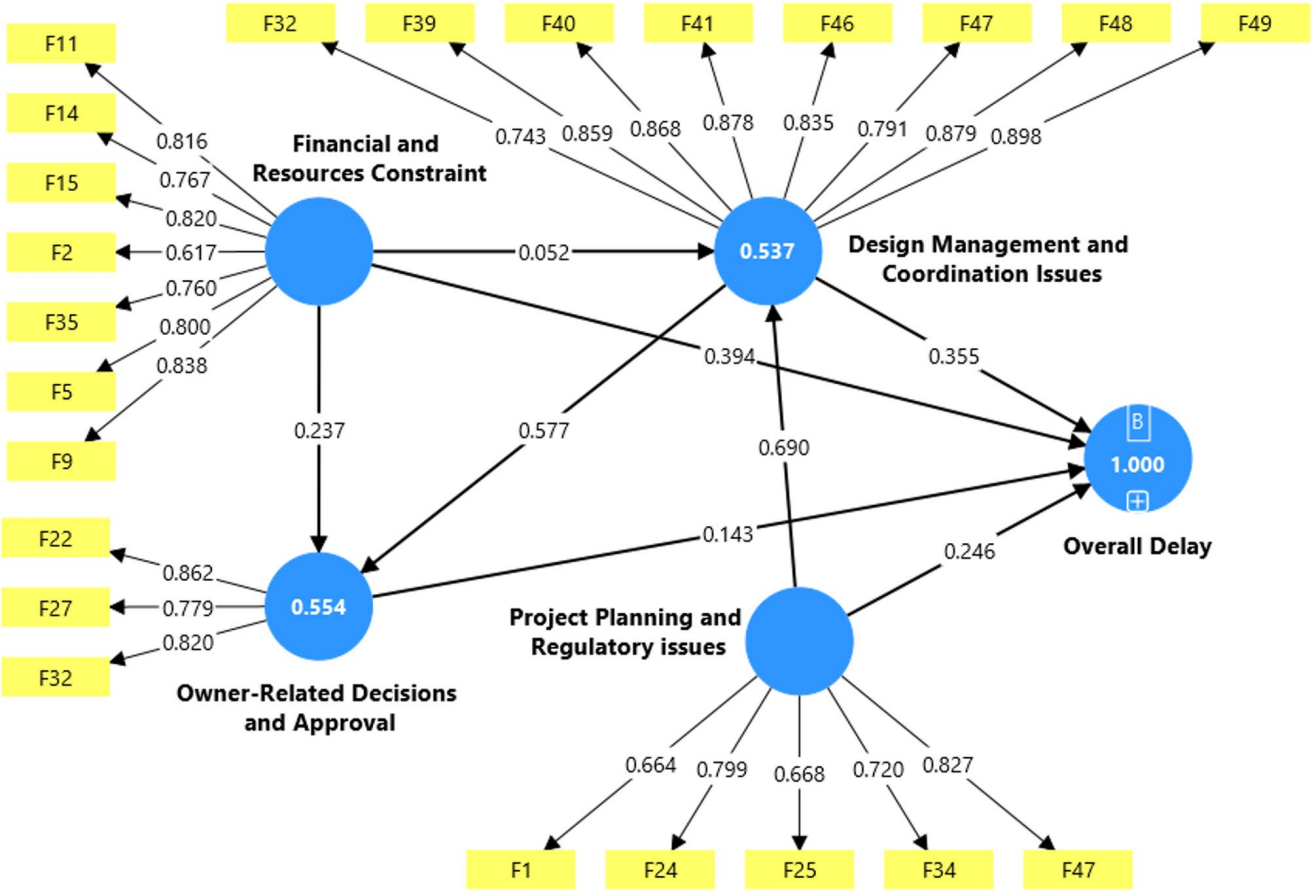
Structural model results.

Design Management and Coordination also significantly affect Owner-Related Decisions (β = 0.57, *p* < 0.001). These findings demonstrate that project delays primarily stem from a chain of deficiencies in planning, design management, and owner decision-making processes. Figure 4 displays the final structural model with path coefficients, highlighting the interrelationship among their collective contribution to the Overall Delay.

## Delay impacts mitigation in Egyptian oil and gas construction projects mitigation

Previous studies have identified managerial and technical practices aimed at reducing delays in oil and gas projects globally. The results of this study have revealed a significant consensus between the solutions proposed by participants and those proposed in previous studies.

Regarding the individual factors (Factors with Full Stakeholder Consensus) extracted from the analysis of RII and Critical Rank (F5, F9, F2), the participants stressed the possibility of addressing these factors by ensuring the availability of the necessary budget, adopting advanced financial mechanisms, and securing the required materials before the start of implementation. This proposal is consistent with previous studies that emphasized securing adequate funding for all project stages, establishing integrated financial systems to monitor payments, and ensuring that materials are available before starting execution operations.

Regarding delays caused by government laws and regulations (F25), participants recommended enhancing transparency, simplifying governmental procedures, and strengthening cooperation and communication among stakeholders using modern technology. These recommendations align with finding from previous studies, which have emphasized the importance of speeding up decision-making process, minimizing bureaucratic complications, and improving coordination between government agencies and contractors to ensure the timely execution of oil and gas construction projects.

Regarding the delay in preparing final designs and obtaining approvals (F40), the participants emphasized the importance of reaching a final agreement on designs among the consultant and owner before starting the execution stage, conducting a comprehensive study of the project, and emphasizing both administrative and design considerations to ensure coherence with timelines. These recommendations are consistent with the results of previous research that confirmed the necessity of completing designs before implementation, improving communication mechanisms between consultants and contractors, and adopting advanced methodologies to ensure the accelerated review of designs and approvals. Table 8 presents a comparison of delay mitigation strategies reported in previous studies and those obtained from the respondents’ views in this study, along with a compatibility range indicating the degree of alignment between them.

**Table 8:** Comparison of delay mitigation strategies between previous studies and current study.

Code	Previous studies	*R*	Participants’ perspective	Compatibility range
F5	1- Utilizing advanced and temporary payments correctly and refraining from diverting project funds for other purposes.	^44^	Ensuring the availability of the required budget before the project starts.	Perfect
	2- The contractor’s dues are paid regularly and promptly according to the actual progress of the work performed.	^45^ and^46^	Developing a quick mechanism for disbursing contractors’ dues and simplifying administrative procedures.	Perfect
	3- 1- An accurate cash flow plan should be optimized and prepared before the start of the project.	^47^	Preparing a comprehensive project study that includes financial, technical, and administrative aspects while assessing potential risks.	Partial
	4- 2- Hiring specialists to enhance cash flow forecasting.	^44^		
	5- Allocating an emergency fund to mitigate potential risks.	^48^	Preparing a quick response plan to address the root causes of delays.	Perfect
	6- Providing the financial plan and schedule using techniques such as Earned Value Management (EVM), The Critical Path Method (CPM), and Project Management Institute (PMI) indicators.	^49^	Establish an accurate and achievable schedule with periodic progress follow-ups.	Perfect
F9	1- Collaborate with subcontractors and suppliers to share risks and develop a comprehensive plan for managing machinery, financial liquidity, materials, and workforce throughout all project phases.	^44^	Preparing a comprehensive project study that includes financial, technical, and administrative aspects while assessing potential risks.	Perfect
	2- Developing a cost- and time-efficient management system model for construction procurement.	^50^	Develop an advanced coordination mechanism among the owner, contractor,and consultant utilizing advanced technology at project stages .	Perfect
F2	1- Enhance the management and control of material supply from the beginning of the project to ensure effective and continuous availability.	^51^	Provide the necessary materials for the project as soon as possible before implementation.	Perfect
	2- Identify the essential materials and equipment that need to be procured and stored in advance from external sources.	^50^		
	3- Develop an integrated plan to manage resources, materials, equipment, and cash flow as essential elements project, ensuring adherence throughout the project stages.	^44^ and ^51^	Preparing a comprehensive project study that includes financial, technical, and administrative aspects while assessing potential risks.	Perfect
	4- Prioritize orders for items with long lead times to minimize the impact of global price increases.	^52^		
	5- Develop a procurement management system model for construction materials and equipment, considering cost and time constraints.	^50^and ^51^	Developing an advanced coordination mechanism among the owner, contractor, and consultant utilizing advanced technology at all project stages.	Perfect
F25	1- Streamline bureaucratic procedures at all stages of project implementation to prevent delays.	^53^	Ensure transparency and minimize government red tape.	Perfect
	2- Obtain the necessary project approvals from the relevant authorities.	^54^		
F40	Consultants must finalize design and construction details before the tender stage to clarify requirements and minimize project risks.	^55^	Predefine the design, tasks, and construction methods.	Perfect

## Study limitations

Despite efforts to ensure the model’s strength and the analysis’s accuracy, the study faces certain limitations. First, the sample size was relatively small compared to studies conducted on general, non-specialized construction projects. Furthermore, restricting the data to specific projects may limit the generalizability of the results. Future research should expand the sample size and test the model in diverse contexts to enhance the reliability and generalizability of the results.

## Conclusion & recommendation

This study systematically classified and analyzed stakeholder-related delay based on their importance, impact, and frequency. The results indicate that selecting a contractor with professional competence and technical and financial experience is a decisive factor in minimizing delays. Streamlining government approval processes for construction permits can significantly shorten project execution timelines. Additionally, hiring specialized consultants with high technical expertise in feasibility studies and design is crucial. This study provides a comprehensive view of the factors affecting delays in Egyptian oil and gas projects, with practical suggestions to enhance performance and achieve project goals within the specified schedule.

Considering the classification and individual analysis of the delay factors, the following practical recommendations can be made:

- Enhance contractors’ financial efficiency and resource management:

Appropriate financial support should be provided to contractors, verifying their financial capability during the contracting process. Contractors should also enhance cash flow management to avoid payment delays to suppliers and subcontractors.

- Develop effective supply chain management:

Utilize the Internet of Things (IoT) to enhance shipment tracking and ensure punctuality.

- Review and simplify government regulations:

The study recommends strengthening cooperation between oil and gas companies and government agencies to streamline procedures and policies related to petroleum construction projects. It also suggests developing electronic platforms to speed up obtaining permits and approvals.

- Contractor selection process:

Owners should establish clear and transparent criteria for selecting contractors, including evaluating financial records, technical expertise, and work experience.

- Accelerate the preparation of designs and approvals:

The study recommends utilizing digital modeling techniques such as “building information modeling systems (BIM)” to improve the speed of preparing designs and executive drawings and obtaining approvals while enhancing cooperation and communication between consultants and stakeholders.

Based on the Structural Equation Model results that showed strong and functional relationships between groups, the most important recommendations can be summarized as follows:

- Adopting an integrated management framework that aligns planning, design, financial coordination, and timely decision-making among all stakeholders.
- Implementation of a detailed execution plan and effective monitoring mechanisms from the project’s early stages.
- Application of a design management and coordination system using Building Information Modeling (BIM).
- Enhancement of communication, reduction of design conflicts, and acceleration of decision processes.
- Assurance of financial stability through proactive planning and emergency reserves.

## Data Availability

The datasets generated and/or analyzed during the current study are available from the corresponding author upon request.
